# Deep and repetitive transcranial magnetic stimulation improves motor dysfunction after basal ganglia infarction: preliminary findings on efficacy and electrophysiological mechanisms

**DOI:** 10.3389/fnins.2026.1870537

**Published:** 2026-06-10

**Authors:** Rui Yang, Yin-Xin Zhu, Xue Chen, Si-Yu Wang, Xiao-Ming Wang

**Affiliations:** Department of Neurology, Affiliated Hospital of North Sichuan Medical College, Nanchong, Sichuan, China

**Keywords:** basal ganglia, cerebral infarction, evoked potential, motor dysfunction, transcranial magnetic stimulation

## Abstract

**Objective:**

To observe the therapeutic effects of deep transcranial magnetic stimulation (dTMS) and repetitive transcranial magnetic stimulation (rTMS) on upper and lower limb motor dysfunction in patients with basal ganglia infarction, and to preliminarily explore their underlying electrophysiological mechanisms.

**Methods:**

Thirty patients with motor dysfunction secondary to basal ganglia infarction, hospitalized at the Affiliated Hospital of North Sichuan Medical College between October 2024 and December 2025, were enrolled in this study. All eligible participants were randomly assigned to one of three treatment groups: dTMS (*n* = 10), rTMS (*n* = 10), or sham stimulation (*n* = 10). All patients in the three groups received routine medical treatment and conventional rehabilitation training. On this basis, the dTMS group was treated with 10 Hz dTMS, the rTMS group with 10 Hz rTMS, and the sham stimulation group with sham stimulation, 5 sessions per week for 2 consecutive weeks. Before treatment, on the first day after treatment, and at 30 days after treatment, the Fugl-Meyer Assessment (FMA), Berg Balance Scale (BBS), and Modified Barthel Index (MBI) were used to evaluate motor function of the affected side and activities of daily living. The resting motor threshold (rMT) and central motor conduction time (CMCT) of the affected hemisphere were measured simultaneously.

**Results:**

The baseline data among the three groups were comparable (all *p* > 0.05); After treatment, there was a statistically significant interaction between group and time in FMA-UE, FMA-LE, MBI, and BBS scores among the three groups (all *p* < 0.05); Compared with baseline, FMA-UE, FMA-LE, MBI, and BBS scores were significantly increased on the first day and at 30 days after treatment in all three groups (all *p* < 0.001); Compared with the sham stimulation group, the dTMS group exhibited higher FMA-UE, FMA-LE, MBI, and BBS scores on the first day and at 30 days after treatment (all *p* < 0.05); Compared with the rTMS group, the dTMS group showed no significant differences in FMA-UE and MBI scores on the first day after treatment (all *p* > 0.05), but higher FMA-LE and BBS scores (all *p* < 0.05), at 30 days after treatment, FMA-UE, FMA-LE, MBI, and BBS scores were all higher in the dTMS group (all *p* < 0.05). There was a statistically significant interaction between group and time in rMT and upper limb CMCT among the three groups after treatment (all *p* < 0.05); Compared with baseline, rMT and upper limb CMCT were significantly decreased on the first day and at 30 days after treatment in all three groups (all *p* < 0.001); Compared with the sham stimulation group, the dTMS group had lower rMT and upper limb CMCT on the first day and at 30 days after treatment (all *p* < 0.05); Compared with the rTMS group, the dTMS group showed lower rMT and upper limb CMCT on the first day after treatment (*p* < 0.05), at 30 days after treatment, rMT was lower (*p* < 0.05), while no significant difference was found in upper limb CMCT (*p* > 0.05).

**Conclusion:**

(1) Both high-frequency dTMS and rTMS can improve upper limb motor dysfunction after basal ganglia cerebral infarction to some extent, and the therapeutic effect of dTMS lasts longer; (2) dTMS has a certain rehabilitative effect on lower limb motor and balance function; (3) The mechanisms underlying the improvement of motor dysfunction after basal ganglia cerebral infarction by high-frequency dTMS and rTMS may be associated with increased excitability of the affected cerebral cortex, enhanced function of the corticospinal tract pathway. In addition, dTMS can directly act on deeper and wider brain regions; (4) Both high-frequency dTMS and rTMS are safe.

## Introduction

Cerebral infarction may cause multiple functional impairments, including disturbances in consciousness, motor ability, cognition, and language (Wang et al., 2025a). The majority of affected individuals suffer from residual dysfunctions of varying degrees, and their rehabilitation remains challenging. Repetitive transcranial magnetic stimulation (rTMS), a non-invasive neuromodulation technique, facilitates neurological recovery following cerebral infarction through multiple mechanisms, including the regulation of synaptic plasticity, the restoration of neurotransmitter homeostasis, and the reorganization of functional networks (Ciricugno et al., 2025; Sharbafshaaer et al., 2024; Kuipers et al., 2025). Meta-analyses have shown that low-frequency rTMS applied to the contralesional hemisphere represents class A evidence for the recovery of upper limb motor function in patients with subacute cerebral infarction, whereas high-frequency rTMS applied to the ipsilesional hemisphere is supported by class B evidence (Lefaucheur et al., 2020). Deep transcranial magnetic stimulation (dTMS), a novel non-invasive neuromodulation technique developed from rTMS, offers the advantages of deeper and broader stimulation (Cheng et al., 2023). Conventional rTMS using a figure-of-eight coil achieves a stimulation depth of approximately 2–3 cm below the skull, whereas dTMS with the H-coil can reach a depth of up to 6 cm and can also engage wider cortical regions without increasing stimulation intensity (Tendler et al., 2016). It has shown considerable potential for the rehabilitation of cerebral infarction involving deep cortical or subcortical regions. Nevertheless, the mechanisms underlying transcranial magnetic stimulation in stroke rehabilitation, particularly its modulation of cortical excitability and associated electrophysiological changes, warrant further investigation. The present study therefore aimed to evaluate the therapeutic effects of dTMS and rTMS on motor dysfunction in patients with basal ganglia infarction and to explore their underlying electrophysiological mechanisms. The innovation of this study lies in the integration of both behavioral assessment and electrophysiological measures. This integration provides new insights into motor rehabilitation strategies for cerebral infarction.

## Methods

### Participants

We randomly assigned participants to one of three groups: dTMS, rTMS, or sham stimulation. Before the intervention, we collected patients’ demographic characteristics (including age, sex, disease duration, and lesion side). We also performed baseline assessments of motor function and electrophysiological parameters on the affected side, covering upper and lower limb motor function, lower limb balance ability, resting motor threshold, and central motor conduction time. Subsequently, a two-week intervention was administered. Outcome reassessments, including affected-side motor function and electrophysiological parameters, were conducted at 1 day and 30 days after the completion of the intervention. The study was carried out at the Affiliated Hospital of North Sichuan Medical College between October 2024 and December 2025. The study protocol was approved by the Ethics Committee of the Affiliated Hospital of North Sichuan Medical College (Approval No. 2025ER716-1), and written informed consent was obtained from all participants prior to enrollment.

Thirty patients with acute basal ganglia infarction were enrolled in this study. Inclusion criteria were as follows: (1) basal ganglia infarction confirmed by cranial CT or MRI with hemiplegia; (2) age 40–80 years and disease duration ≤2 weeks; (3) alert mental status without severe cognitive dysfunction; (4) signed informed consent. Exclusion criteria were: (1) severe cardiac, pulmonary, or systemic illness precluding treatment; (2) history of intracranial hemorrhage or epilepsy; (3) TMS contraindications (e.g., intracranial metal objects, cardiac pacemaker); (4) concurrent infection, fracture, or other conditions affecting the affected limb.

### Interventions

Patients in all three groups received standard care and routine rehabilitation training for cerebral infarction. In addition, they were treated with transcranial magnetic stimulation (TMS) according to the following protocols. dTMS group: An M-100 Ultimate deep transcranial magnetic stimulator (Brainsway Ltd., Jerusalem, Israel) fitted with an HF001A H7 coil was used for treatment. rTMS group: An M-100 Ultimate transcranial magnetic stimulator (Brainsway Ltd., Jerusalem, Israel) equipped with a BY90A figure-of-eight coil was used. Sham group: Patients in the sham group received sham stimulation using the same figure-of-eight coil as in the rTMS group, with no effective magnetic field generated. Stimulation protocol: The stimulation target was the ipsilesional primary motor cortex (M1). High-frequency (10 Hz) stimulation was selected because it has been shown to increase cortical excitability of the stimulated hemisphere and promote motor recovery after stroke; the ipsilesional M1 was chosen as the target based on the interhemispheric competition model, which suggests that enhancing excitability of the affected hemisphere helps rebalance interhemispheric inhibition (Su et al., 2025). Parameters were set as follows: frequency, 10 Hz; intensity, 100% of the resting motor threshold (rMT); train duration, 5 s; inter-train interval, 20 s; total pulses per session, 3,600. Each session lasted 30 min and was administered once daily, five days per week, for two consecutive weeks.

### Outcome measures

Motor function of the affected side was assessed using the Fugl-Meyer Assessment (FMA), which comprises the upper extremity subscale (FMA-UE) and the lower extremity subscale (FMA-LE). The FMA-UE consists of 33 items, each scored from 0 to 2, yielding a maximum total score of 66 points; higher scores indicate better upper limb motor function. The FMA-LE consists of 17 items, each also scored from 0 to 2, with a maximum total of 34 points; higher scores reflect better lower limb function.

Balance function was quantitatively evaluated using the Berg Balance Scale (BBS), which includes 14 items, each rated from 0 to 4, with a maximum total score of 56 points. A higher score indicates better balance performance. The modified Barthel Index (MBI) was used to assess patients’ ability to perform activities of daily living. The scale comprises 10 items, with a maximum total score of 100 points. A higher score indicates a greater level of independent living ability. The combined use of FMA (upper/lower limb motor function), BBS (balance), and MBI (activities of daily living) is commonly used in stroke rehabilitation research. These scales are complementary: FMA captures motor impairment severity, BBS evaluates postural control, and MBI reflects real-world functional independence. Together, they provide a comprehensive, clinically meaningful profile of post-stroke functional recovery. For the measurement of resting motor threshold (rMT) and central motor conduction time (CMCT), the procedure was as follows: First, the optimal scalp position (hotspot) for eliciting motor evoked potentials (MEPs) from the contralateral first dorsal interosseous muscle was identified over the ipsilesional M1 hand area. rMT was defined as the lowest stimulus intensity that produced MEPs >50 μV in at least 5 of 10 consecutive trials. Next, with stimulation intensity set at 80% of rMT at the hotspot, five reproducible MEP waveforms with the largest amplitudes were selected, and their average latency was recorded as cortical latency. The stimulation site was then moved to the paraspinal region adjacent to the C7 spinous process on the affected side, keeping all other settings unchanged, and the obtained latency was defined as spinal latency (Tekgul et al., 2020). CMCT was calculated as the difference between cortical latency and spinal latency.

### Blinding and randomization

This was a single-blind, randomized controlled trial. This study was designed as an exploratory investigation to generate preliminary data on the efficacy and safety of dTMS versus rTMS in basal ganglia infarction. In accordance with common practice for small-scale exploratory studies in neuromodulation research and based on feasibility considerations, no formal power calculation was performed. The target sample size was set at 8–10 participants per group, and a total of 28 participants completed the study protocol. An independent researcher generated a computer-based random sequence and concealed the random numbers in sequentially numbered, opaque envelopes. The envelopes were opened in numerical order by a designated researcher, who then administered the interventions. A total of 28 participants were randomly assigned to three groups: dTMS, rTMS, or sham stimulation. Participants, outcome assessors, and statistical analysts were blinded to group allocation, whereas the treating researcher was not blinded because the stimulation protocols required different coils and settings.

### Statistical analysis

Statistical analyses were carried out using IBM SPSS Statistics version 26.0 (IBM Corp., Armonk, NY, USA). A two-tailed significance level of *α* = 0.05 was adopted, and *p* < 0.05 was regarded as indicating statistical significance. The Shapiro–Wilk test was used to examine the normality of the data. Normally distributed continuous variables are presented as mean ± standard deviation ( $\bar{x}\pm s$ ). We compared baseline continuous data among the three groups using one-way ANOVA. Between-group differences were analyzed using repeated-measures ANOVA. The sphericity assumption was evaluated with Mauchly’s test; if sphericity was rejected, the Greenhouse–Geisser correction was applied. When a significant group-by-time interaction was detected, simple effects analyses were performed as *post hoc* comparisons. For within-group comparisons, the Bonferroni method was used to adjust for multiple comparisons. Categorical variables are reported as counts (*n*), and between-group comparisons were made using Fisher’s exact test.

## Results

### Study participation

**Figure 1:**
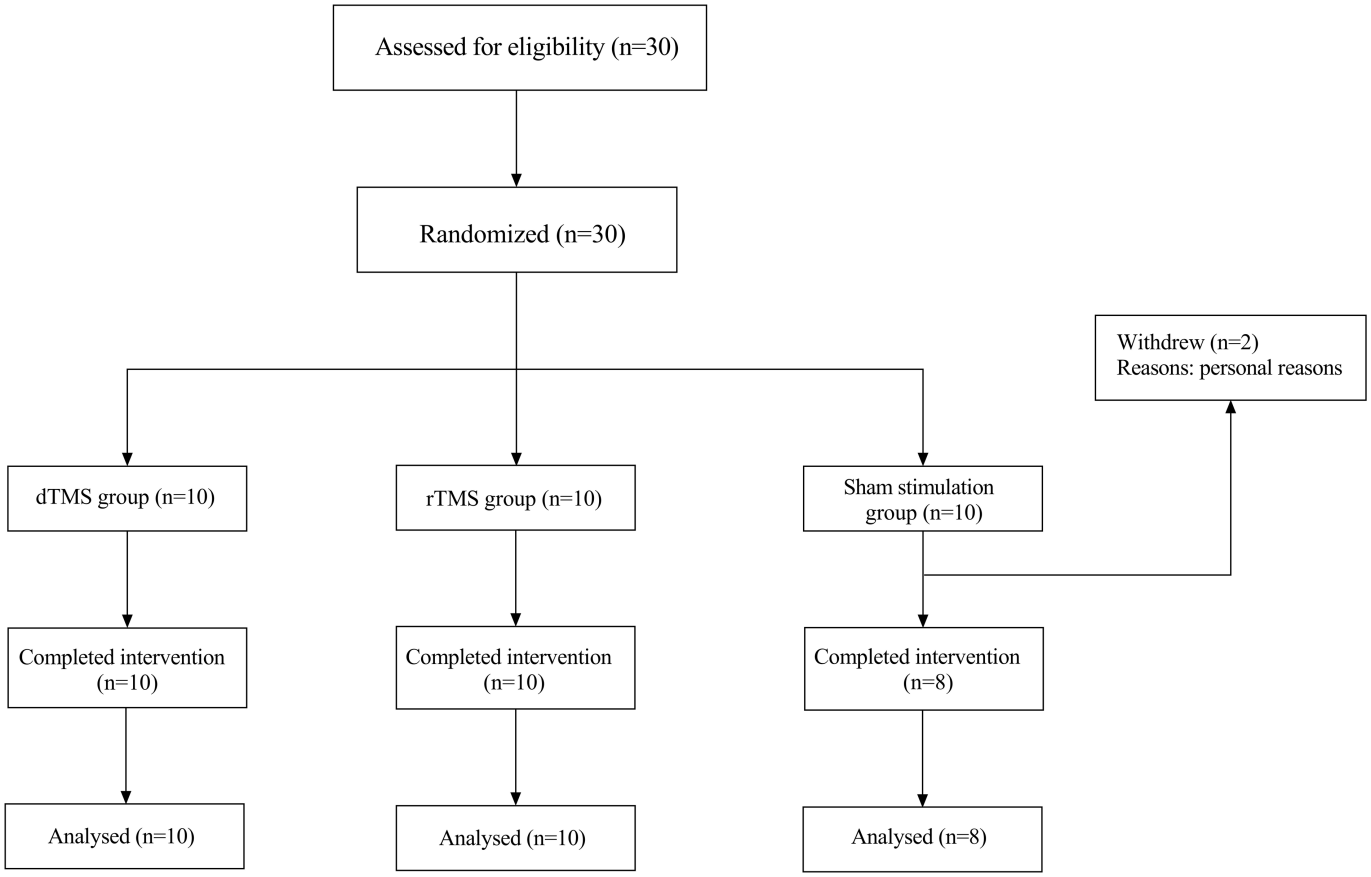
Reasons for not receiving influenza vaccination in 2024.

A total of 30 patients provided written informed consent and were enrolled in this study. Two patients in the sham stimulation group withdrew from the trial due to personal reasons before completing the intervention. Consequently, 28 patients completed the entire study protocol and were included in the final analysis: 10 patients in the dTMS group, 10 in the rTMS group, and 8 in the sham stimulation group (Figure 1).

Specifically, the dTMS group included 7 males and 3 females, with a mean age of 55.60 ± 5.81 years. The rTMS group included 6 males and 4 females, with a mean age of 56.20 ± 7.67 years. The sham stimulation group included 4 males and 4 females, with a mean age of 57.38 ± 5.09 years. No significant intergroup differences were observed in any baseline demographic or clinical characteristics (all *p* > 0.05; Table 1). During the treatment period, only two patients in the dTMS group reported transient dizziness, which resolved spontaneously with rest. No other adverse effects were reported in any patient. During the 30-day follow-up period, no adverse events were observed in any of the three groups. No significant intergroup differences were found in adverse event rates or safety profiles.

**Table 1:** Demographic characteristics at baseline.

Stimulation methods	dTMS group (*n* = 10)	rTMS group (*n* = 10)	Sham group (*n* = 8)	*p* value
Age, (years)	55.60 ± 5.81	56.20 ± 7.67	57.38 ± 5.09	0.840
Gender, *n*				0.885
Male	7	6	4	
Female	3	4	4	
Course of the disease, (days)	7.60 ± 1.57	7.70 ± 2.05	7.63 ± 1.76	0.992
Lesion side, *n*				1.000
Left	4	4	3	
Right	6	6	5	
HR (bpm)	83.50 ± 7.70	84.60 ± 7.70	84.63 ± 8.45	0.939
SBP (mmHg)	140.50 ± 9.25	144.00 ± 9.28	140.38 ± 8.92	0.622
DBP (mmHg)	86.90 ± 9.01	91.50 ± 6.32	85.88 ± 8.72	0.290
Drinking, *n*	3	4	4	0.885
Smoking, *n*	7	8	4	0.526
Comorbidities				
Hypertension, *n*	8	8	8	0.512
Hyperlipemia, *n*	6	6	5	1.000
Diabetes, *n*	4	3	3	1.000

### Upper and lower extremity motor ability

A two-way repeated measures ANOVA was conducted to compare FMA-UE scores among the three groups across different time points. The analysis revealed a statistically significant group effect (*F* = 4.184, *p* < 0.05), a significant time effect (*F* = 834.063, *p* < 0.001), and a significant group × time interaction (*F* = 26.963, *p* < 0.001). Further *post hoc* comparisons indicated that, relative to pre-treatment baseline, all three groups demonstrated marked improvements in FMA-UE scores at both 1 day and 30 days following the intervention (*p* < 0.001). When compared to the sham stimulation group, the dTMS group achieved significantly higher FMA-UE scores at both post-treatment time points (*p* < 0.05). In comparison with the rTMS group, the dTMS group showed no significant difference in FMA-UE score at 1 day post-treatment (*p* > 0.05); however, at 30 days post-treatment, the dTMS group exhibited a significantly higher score (*p* < 0.05). The complete results are summarized in Table 2.

**Table 2:** Comparison of FMA-UE and FMA-LE scores among the three groups at baseline, 1 day, and 30 days after treatment.

Group	Time point	FMA-UE score (mean ± SD)	FMA-LE score (mean ± SD)
dTMS	Pre-treatment	38.80 ± 3.01	18.10 ± 3.03
	1 day post-treatment	53.90 ± 3.47^a^	26.40 ± 2.17^a^^,^^b^
	30 days post-treatment	59.50 ± 2.99^a^^,^^b^	29.50 ± 2.44^a^^,^^b^
rTMS	Pre-treatment	38.60 ± 2.79	19.10 ± 3.17
	1 day post-treatment	53.10 ± 2.07^a^	23.00 ± 2.82
	30 days post-treatment	55.70 ± 1.82	26.20 ± 2.37
Sham	Pre-treatment	39.88 ± 3.38	19.13 ± 5.27
	1 day post-treatment	47.38 ± 5.04	23.00 ± 2.50
	30 days post-treatment	50.88 ± 5.59	25.88 ± 2.09

a p<0.05 vs. sham stimulation group at the same time point.

b p<0.05 vs. rTMS group at the same time point. Data are presented as mean ± standard deviation (SD).

A two-way repeated measures ANOVA was conducted to compare FMA-LE scores among the three groups across different time points. The analysis revealed a statistically significant group effect (*F* = 6.807, *p* < 0.05), a significant time effect (*F* = 289.929, *p* < 0.001), and a significant group × time interaction (*F* = 12.196, *p* < 0.001). *Post hoc* comparisons indicated that, relative to pre-treatment baseline, all three groups showed marked improvements in FMA-LE scores at both 1 day and 30 days following the intervention (*p* < 0.001). When compared to the sham stimulation group, the dTMS group achieved significantly higher FMA-LE scores at both post-treatment time points (*p* < 0.05). Likewise, compared with the rTMS group, the dTMS group exhibited significantly higher FMA-LE scores at 1 day and 30 days post-treatment (*p* < 0.05). Complete results are summarized in Table 2.

### Activities of daily living

A two-way repeated measures ANOVA was performed to compare modified Barthel Index (MBI) scores among the three groups across different time points. The analysis yielded a significant main effect of group (*F* = 4.439, *p* < 0.05), a significant main effect of time (*F* = 617.264, *p* < 0.001), and a significant group × time interaction (*F* = 24.066, *p* < 0.001). *Post hoc* analyses revealed that, compared with baseline, all three groups exhibited significant increases in MBI scores at 1 day and 30 days after treatment (*p* < 0.001). Relative to the sham stimulation group, the dTMS group had significantly higher MBI scores at both follow-up time points (*p* < 0.05). In comparison with the rTMS group, the dTMS group showed no significant difference in MBI score at 1 day post-treatment (*p* > 0.05); however, at 30 days post-treatment, the dTMS group demonstrated a significantly higher score (*p* < 0.05). Detailed results are presented in Table 3.

**Table 3:** Comparison of MBI and BBS scores among the three groups at baseline, 1 day, and 30 days after treatment.

Group	Time point	MBI (points)	BBS (points)
dTMS	Pre-treatment	43.70 ± 8.80	31.00 ± 5.90
	1 day post-treatment	64.20 ± 7.59^a^	41.30 ± 4.66^a^^,^^b^
	30 days post-treatment	78.80 ± 8.13^a^^,^^b^	47.30 ± 4.71^a^^,^^b^
rTMS	Pre-treatment	43.50 ± 6.29	30.80 ± 7.72
	1 day post-treatment	62.20 ± 8.36^a^	36.70 ± 7.15
	30 days post-treatment	69.90 ± 7.65	38.70 ± 7.82
Sham	Pre-treatment	43.88 ± 6.61	31.25 ± 7.45
	1 day post-treatment	53.25 ± 4.74	34.50 ± 7.09
	30 days post-treatment	61.38 ± 5.15	37.87 ± 6.97

a p<0.05 vs. sham stimulation group at the same time point.

b p<0.05 vs. rTMS group at the same time point. Data are presented as mean ± standard deviation (SD).

### Balance function

A two-way repeated measures ANOVA was performed to compare Berg Balance Scale (BBS) scores among the three groups across different time points. The analysis yielded a significant main effect of group (*F* = 6.873, *p* < 0.05), a significant main effect of time (*F* = 565.155, *p* < 0.001), and a significant group × time interaction (*F* = 61.292, *p* < 0.001). *Post hoc* analyses revealed that, compared with baseline, all three groups exhibited significant increases in BBS scores at 1 day and 30 days after treatment (*p* < 0.001). Relative to the sham stimulation group, the dTMS group had significantly higher BBS scores at both follow-up time points (*p* < 0.05). In comparison with the rTMS group, the dTMS group also demonstrated significantly higher BBS scores at both 1 day and 30 days post-treatment (*p* < 0.05). Detailed results are presented in Table 3.

### Neurophysiological measures

A two-way repeated measures ANOVA was performed to compare ipsilesional resting motor threshold (rMT) among the three groups across different time points. The analysis yielded a significant main effect of group (*F* = 10.654, *p* < 0.05), a significant main effect of time (*F* = 199.168, *p* < 0.001), and a significant group × time interaction (*F* = 5.137, *p* < 0.05). *Post hoc* analyses revealed that, compared with baseline, all three groups exhibited significant reductions in ipsilesional rMT at 1 day and 30 days after treatment (*p* < 0.001). Relative to the sham stimulation group, the dTMS group had significantly lower ipsilesional rMT values at both follow-up time points (*p* < 0.05). In comparison with the rTMS group, the dTMS group showed no significant difference in ipsilesional rMT at 1 day post-treatment (*p* > 0.05); however, at 30 days post-treatment, the dTMS group demonstrated a significantly lower rMT (*p* < 0.05). Detailed results are presented in Table 4.

**Table 4:** Comparison of rMT and CMCT among the three groups at baseline, 1 day, and 30 days after treatment.

Group	Time point	rMT (%)	CMCT (ms)
dTMS	Pre-treatment	41.20 ± 3.52	9.79 ± 2.28
	1 day post-treatment	32.40 ± 1.77^a^	8.58 ± 0.48^a^^,^^b^
	30 days post-treatment	32.00 ± 1.24^a^^,^^b^	8.49 ± 0.45^a^
rTMS	Pre-treatment	42.10 ± 2.99	9.80 ± 0.18
	1 day post-treatment	34.20 ± 1.39^a^	8.62 ± 0.42^a^
	30 days post-treatment	36.00 ± 1.76	8.59 ± 0.43^a^
Sham	Pre-treatment	41.00 ± 3.29	9.68 ± 0.43
	1 day post-treatment	37.25 ± 2.43	9.33 ± 0.34
	30 days post-treatment	36.50 ± 2.50	9.16 ± 0.31

a p<0.05 vs. sham stimulation group at the same time point.

b p<0.05 vs. rTMS group at the same time point. Data are presented as mean ± standard deviation (SD).

A two-way repeated measures ANOVA was performed to compare ipsilesional upper limb central motor conduction time (CMCT) among the three groups across different time points. The analysis yielded a significant main effect of group (*F* = 4.061, *p* < 0.05), a significant main effect of time (*F* = 237.902, *p* < 0.001), and a significant group × time interaction (*F* = 16.79, *p* < 0.05). *Post hoc* analyses revealed that, compared with baseline, all three groups exhibited significant shortening of ipsilesional upper limb CMCT at 1 day and 30 days after treatment (*p* < 0.001). Relative to the sham stimulation group, the dTMS group had significantly shorter ipsilesional upper limb CMCT at both follow-up time points (*p* < 0.05). In comparison with the rTMS group, the dTMS group showed lower ipsilesional upper limb CMCT at 1 day post-treatment (*p* < 0.05); however, at 30 days post-treatment, no statistically significant difference was observed in ipsilesional upper limb CMCT (*p* > 0.05). Detailed results are presented in Table 4.

## Discussion

In recent years, although positive progress has been made in the treatment of acute cerebral infarction, a considerable number of patients still develop varying degrees of motor dysfunction, and the recovery of upper and lower limb paralysis remains heterogeneous (Zhang et al., 2024). A series of studies have shown that high-frequency dTMS can improve upper and lower limb motor dysfunction in patients with cerebral infarction, and these beneficial effects can be maintained for a relatively long period (Chieffo et al., 2014; Chieffo et al., 2021; Chieffo et al., 2018). Su et al. reported that 3 weeks of 10 Hz dTMS applied to the ipsilesional primary motor cortex (M1) during the recovery phase of cerebral infarction and cerebral hemorrhage had a favorable rehabilitative effect on lower limb motor function (Su et al., 2025). Wang et al. demonstrated that both 5 Hz dTMS and rTMS could improve lower limb motor function in patients with subacute stroke, with dTMS showing a more pronounced pro-recovery effect (Su et al., 2025). Furthermore, Zhang et al. reported that dTMS was superior to rTMS in improving balance and gait function in stroke patients (Wang et al., 2025b).

In this study, patients with motor dysfunction following basal ganglia infarction were randomly assigned to receive high-frequency (10 Hz) dTMS, rTMS, or sham stimulation. Motor function and activities of daily living were assessed before treatment, as well as at 1 day and 30 days after treatment. The results demonstrated that FMA-UE, FMA-LE, MBI, and BBS scores in all three groups were significantly higher after treatment compared with baseline. Relative to the sham stimulation group, the dTMS group exhibited significantly higher scores on all four measures at both post-treatment time points. Compared with the rTMS group, the dTMS group showed significantly higher FMA-LE and BBS scores at 1 day post-treatment, and significantly higher scores on all four measures at 30 days post-treatment. These findings suggest that both dTMS and rTMS effectively improve upper limb motor dysfunction, whereas dTMS demonstrates superior effects on lower limb motor function and balance, with a longer duration of therapeutic benefit. These results are consistent with the findings of the aforementioned studies.

The prevailing theory regarding motor recovery after cerebral infarction is the interhemispheric competition model. These theory posits that the normally balanced interhemispheric interaction mediated by the corpus callosum is disrupted following cerebral infarction, resulting in reduced inhibition from the affected hemisphere to the unaffected hemisphere and enhanced inhibition from the unaffected hemisphere to the affected hemisphere (Zhang et al., 2025). In basal ganglia infarction, the cortico-basal ganglia-thalamo-cortical loop—a key subcortical circuit that regulates cortical excitability—is directly involved, potentially leading to more pronounced hypoexcitability of the ipsilesional M1. This pathological feature underscores the need for a stimulation technique that can effectively reach deep structures to restore interhemispheric balance. Repetitive transcranial magnetic stimulation (rTMS) can enhance cortical excitability on the affected side and improve corticospinal tract function by modulating synaptic plasticity, including long-term potentiation (Di Pino et al., 2014; Okamoto et al., 2021; Hoonhorst et al., 2021). As a key fiber tract for motor function, the corticospinal tract originates primarily from the primary motor cortex (M1), and its functional status directly influences motor recovery (Deng et al., 2025; Diao et al., 2017). In clinical practice, high-frequency (≥5 Hz) rTMS or dTMS is often applied to increase excitability of the ipsilesional M1, whereas low-frequency (≤1 Hz) stimulation is used to suppress excessive activation of the contralesional hemisphere, thereby reestablishing interhemispheric balance (Zhao et al., 2025). Conventional rTMS achieves a stimulation depth of approximately 2–3 cm below the skull, whereas dTMS can reach a depth of about 6 cm below the skull, directly targeting deep cortical and subcortical structures without requiring an increase in stimulation intensity (Sun and Yu, 2026). Theoretically, dTMS should produce superior effects compared with rTMS; however, systematic reports on the mechanisms underlying the effects of these two stimulation modalities on motor dysfunction after cerebral infarction, particularly with respect to electrophysiological aspects, remain lacking.

Most of the cited literature in the above paragraphs focuses on limb motor function outcomes. To bridge the gap between behavioral recovery and neurophysiological changes, we further explored electrophysiological indicators. The present study further explored the electrophysiological mechanisms associated with dTMS and rTMS. The results showed that at both 1 day and 30 days after treatment, ipsilesional rMT and upper limb CMCT were reduced compared with baseline in all three groups. Relative to the sham stimulation group, the dTMS group exhibited lower values for both measures at the two post-treatment time points. Compared with the rTMS group, the dTMS group showed lower ipsilesional rMT and upper limb CMCT at 1 day post-treatment. However, at 30 days post-treatment, only ipsilesional rMT remained significantly lower in the dTMS group, while no statistically significant difference was observed in upper limb CMCT between the two groups. These findings suggest that both dTMS and rTMS can enhance ipsilesional cortical excitability and shorten CMCT to a certain extent, with dTMS demonstrating a longer duration of therapeutic effect. The underlying mechanism may be related to the ability of dTMS to directly target deeper and broader brain regions, thereby producing more direct and extensive stimulation. This possibility warrants further investigation.

In summary, both high-frequency dTMS and rTMS can improve upper limb motor dysfunction following basal ganglia infarction to a certain extent, while dTMS additionally exerts rehabilitative effects on lower limb motor function and balance, with more sustained therapeutic benefits. The underlying mechanisms may involve the enhancement of ipsilesional cortical excitability, improvement of corticospinal tract function, facilitation of neural remodeling, and the modulatory effects of dTMS on deep brain regions. The present study has several limitations, including a relatively small sample size and the absence of long-term follow-up. In addition, as a single-center study, the generalizability of our findings may be limited. Future research should consider expanding the sample size or conducting large-scale, multicenter longitudinal studies to further evaluate the efficacy and safety of dTMS alone or in combination with rTMS for motor dysfunction after cerebral infarction. In addition, more precise neuromodulation strategies could be achieved by integrating multimodal neuroimaging and neurophysiological techniques such as electroencephalography (EEG), and further investigations into the underlying mechanisms are warranted.

## Data Availability

The raw data supporting the conclusions of this article will be made available by the authors, without undue reservation.
